# “Parasite-induced aposematism” protects entomopathogenic nematode parasites against invertebrate enemies

**DOI:** 10.1093/beheco/arv202

**Published:** 2015-11-27

**Authors:** Rebecca S. Jones, Andy Fenton, Michael P. Speed

**Affiliations:** Department of Evolution, Ecology and Behaviour, Institute of Integrative Biology, University of Liverpool, Crown Street, Liverpool L69 7ZB, Merseyside, UK

**Keywords:** aposematism, communication, host–parasite, predation, signaling.

## Abstract

Aposematism is a well-known strategy in which prey defend themselves from predation by pairing defenses such as toxins, with warning signals that are often visually conspicuous color patterns. Here, we examine the possibility that aposematism can be induced in a host by colonies of infectious parasites in order to protect the parasites from the consequences of attacks on the host. Earlier studies show that avian predators are reluctant to feed on carcasses of host prey that are infected with the entomopathogenic nematode, *Heterorhabditis bacteriophora*. As the age of infection increases, the parasites kill and preserve the host and subsequently cause its color to change, becoming bright pink then red. Nematode colonies in dead hosts may also be vulnerable, however, to nocturnally active foragers that do not use vision in prey detection. Here, then we test a novel hypothesis that the nematode parasites also produce a warning odor, which functions to repel nocturnally active predators (in this case, the beetle *Pterostichus madidus*). We show that beetles decrease their feeding on infected insect prey as the age of infection increases and that olfactory cues associated with the infections are effective mechanisms for deterring beetle predation, even at very early stages of infection. We propose that “parasite-induced aposematism” from the nematodes serves to replace the antipredator defenses of the recently killed host. Because sessile carcasses are exposed to a greater range of predators than the live hosts, several alternative defense mechanisms are required to protect the colony, hence aposematic signals are likely diverse in such “parasite-induced aposematism.”

## Lay Summary

Parasites can manipulate their hosts to ward off predators, by making them glow, become smelly, and toxic. We show this experimentally. Odors protect young infections particularly well.

## INTRODUCTION

Parasite-induced alteration of host phenotype is a widespread strategy of transmission among pathogens (Moore 2002). Many parasites manipulate their host’s behavior or coloration to maximize transmission to a definitive host by making the intermediate host more conspicuous to predators, the definitive host (Moore 2002). For example, ants infected with the trematode *Dicrocoelium dendriticum* move up to the top of vegetation, increasing their chance of being eaten by grazing sheep, the definitive host (Moore 1995). Thus, the parasite increases its chance of transmission by increasing the likelihood of the intermediate host being consumed by the definitive host species. However, some parasites only have 1 host in their life cycle and as a result, predation of this host can be detrimental to the parasite if it is unable to survive and reproduce within the predator. Here, we demonstrate a novel form of odor-based host manipulation by a parasitic nematode in order to deter predators from consuming an infected host, protecting the nematode–bacterium colony within.

Entomopathogenic nematodes (EPNs, obligate insect parasites) infect and kill insect hosts. They make use of an obligate bacterial symbiont that first kills the insect host and then suppresses the growth of microbial competitors, preventing the host carcass from decomposition (Waterfield et al. 2009). A well-studied example of this symbiosis is the EPN, *Heterorhabditis bacteriophora* (Nematoda, Rhabditidae) and its symbiotic bacterium, *Photorhabdus luminescens* (Clarke 2008; Waterfield et al. 2009; Dillman et al. 2012), which infect a large range of soil-dwelling insects. As with other EPNs, there is an incubation period between initial infection and release of infectious juvenile forms into the surrounding soil to find new hosts. For *H. bacteriophora* and *P. luminescens*, this incubation period may be as long as 20 days (Clarke 2008). If foraging animals attack and consume the host carcass during the incubation period, they will ingest the entire colony. Ingested nematodes are very unlikely to survive in the predator’s gut, and are not known to infect the predator (Fenton et al. 2011). Hence, ingestion is very likely terminal for the colony. A key, but underexplored, question in understanding the biology of EPNs is then how colonies protect themselves from such a fatal attack by foraging animals during this prolonged period of vulnerability.

Recently, Fenton et al. (2011) proposed a novel hypothesis that we term “parasite-induced aposematism” as the key strategy in colony defense. In aposematism, a chemical defense, such as a toxin, is associated with a warning signal such as a conspicuous color pattern seen in many toxic species (e.g., ladybirds *Coccinella septempunctata*) or venomous species such as many wasps and bees (Mappes et al. 2005). A conspicuous color pattern is easier for a predator to detect against a background, but it is also easier to learn and remember (Roper 1990). This effect is then further enhanced by the presence of the chemical defense (Guilford 1990; Gamberale-Stille and Guilford 2004; Skelhorn and Rowe 2006; Holen and Svennungsen 2012). Fenton et al. (2011) proposed that the nematode and its symbiotic bacterium protect their host’s carcass by causing it to manifest aposematic traits.

In support of this “parasite-induced aposematism” hypothesis, colonies of several species of EPN, including *H. bacteriophora* are known to confer chemical defense on host carcasses, repelling species of ant (Baur et al. 1998; Gulcu et al. 2012), beetles (Foltan and Puza 2009), crickets, and wasps (Gulcu et al. 2012). Host carcasses infected with *H. bacteriophora* are known to be protected through repellent metabolic products of its bacterial symbiont (Zhou et al. 2002; Clarke 2008). In *P. luminescens*, an insecticidal protein toxin complex is secreted after insect death (toxin complex A, “Tca”), which is known to kill or delay growth of insects, including the Colorado potato beetle, *Leptinotarsa decemlineata*, and the sweet potato fly, *Bemisia tabaci* (Blackburn et al. 2005). Therefore, the orally toxic Tca is likely targeted toward foraging scavangers such as ants and other soil-dwelling predators (Daborn et al. 2001; Waterfield et al. 2009). Hence, one component of aposematism, chemical defense, is clearly present in EPNs and its molecular basis is sometimes known.

Fenton et al. (2011) also argued that the second component of aposematism, conspicuous warning coloration is also present in infected carcasses. In *H. bacteriophora* infections, there is a transient period of host bioluminescence between 24 and 36h after infection, which is conferred by the bacterium (but not in other EPNs that lack *P. luminescens*) (Waterfield et al. 2009). This could conceivably act as an aposematic cue. However, in *H. bacteriophora* and commonly in other EPNs, there is a longer lasting color change to the host’s epidermis, which, in *H. bacteriophora*, goes through orange to bright pink–red after 7 days. This pigment is also produced bacterially (Clarke 2008). Fenton et al. (2011) demonstrated that European robins (*Erithacus rubecula*) were significantly less likely to handle or consume waxworms (*Galleria mellonella* larvae) that had changed color after infection by *H. bacteriophora* compared with uninfected individuals.

Though parasite-induced warning coloration seems a likely explanation, it is in our view unlikely to be the whole story of colony defense in EPNs. Warning coloration is, for example, unlikely to protect prey from nocturnally active soil-dwelling predators such as beetles and spiders that have poor vision and operate in low levels of ambient light. Without a warning cue, these foragers could cause damage to the carcass and injure the colony within it before being repelled by the chemical defense. Hence, we argue that an alternative, nonvisual first line of defense is likely to deter nonvisual predators or those foraging at night. When culturing *H. bacteriophora* in the laboratory, we noted a pungent odor associated with infections (and not with uninfected, decaying carcasses) and hypothesized that this odor might act as an aposematic cue in itself, repelling and causing wariness in nocturnally active predators (Eisner and Grant 1981). We investigate whether this olfactory cue can function as an aposematic cue.

A second point of interest is that colony defenses are not necessarily produced instantaneously with infection. Rather the epidermal color changes take several days to develop (e.g., Fenton et al. 2011), and conceivably, this may be the case with protective toxins too (see Gulcu et al. 2012). Hence, we hypothesized that olfactory aposematism might be in place more rapidly than color and toxicity changes, providing an early line of defense, while the other components of aposematism build up.

Here, then we test this hypothesis of olfactory infectious aposematism with experiments using nocturnal, soil-dwelling beetles (*Pterostichus madidus*, Coleoptera, Carbidae) as predators. We sought to investigate the dynamics of chemical and aposematic defenses with *H. bacteriophora* infections, measuring changes in protection associated with changes in phenotypes over time.

We performed 2 experiments to test these hypotheses: the first examined feeding-related behaviors of a nocturnally active, nonvisually hunting forager (the beetle *P. madidus*) (Wheater 1989) in relation to infected or uninfected waxworms; the second, the effect of infected or uninfected waxworm odor on the beetles.

## METHODS

### Beetle collection and housing

Ground beetles (Coleoptera: Carabidae) were trapped in pitfall traps located in a small wooded area at Dale Hall of Residence (University of Liverpool, Mossley Hill, Liverpool, UK). Seven unbaited traps were set up in a transect 1 m apart using plastic tumblers with a diameter of 7cm, with a 20×12cm^2^ cardboard cover. Trapping ran from 1 July 13 to 5 August 13 and from 19 May 14 to 3 September 14, and ground beetles (henceforth beetles) were collected from traps every 3 days. Manual foraging, that is, turning over logs was carried out at Ness Gardens (Neston, Wirral) on 03 July 13. In 2013, 38 *P. madidus* were caught and in 2014, 62 *P. madidus* were caught. Beetles were sexed after both experiments. Data were pooled across both years because there was no effect of year on time spent feeding (Markov chain Monte Carlo generalized linear mixed model [MCMCglmm], *P* = 0.726), time spent in the target area (MCMCglmm, *P* = 0.634) or time spent on a scent (MCMCglmm, *P* = 0.988). Experimental setup and housing was consistent across both years.

Beetles were housed in individual rectangular containers (Smart Tubz, Tesco, 11cm × 16cm × 4.5cm) with circa 2cm of soil, small twigs (for hiding), and dog food (Cesar’s country chicken and vegetable) was provided ad libitum as food. Beetles were also sprayed weekly with a hand-operated plant mister and were kept under a photoperiod of 18:6 L:D at 20±1 °C. Beetles were given 7 days to acclimatize to the photoperiod and surroundings before any experiments commenced and allowed a further week between experiments. A total of 53 male and 27 female beetles were utilized in all the experiments and were sexed when dissected following trials (Supplementary Material S1).

## EXPERIMENT 1: EFFECT OF NEMATODE–BACTERIUM INFECTION ON PREDATION BY GROUND BEETLES

To test whether nematode-infected carcasses have protection against invertebrate foragers, we presented individual beetles with a single, waxworm larva in a small behavioral arena and recorded their behaviors in relation to larva that were either infected or uninfected.

### Waxworm infection

Waxworms (*G. mellonella*) were infected with *H. bacteriophora* (strain TTO1 supplied by D. Clarke and S. Joyce from University College Cork) using standard techniques in which 10 waxworms were placed on filter paper with 1000 IJs/mL of nematode culture in a 90-mm petri dish (Kaya and Stock 1997). Waxworms were then frozen 3, 5, and 7 days postinfection along with fresh uninfected waxworms. Each beetle was used for 2 trials, one with an infected waxworm of a specified stage of infection and one with an uninfected waxworm. Order of presentation was systematically randomized so that, for example, 15 beetles had an infected waxworm first, whereas 15 received the uninfected waxworm first. We left at least 7 days between presentations. We aimed for 15 beetles in each subgroup, but deaths of some animals left the subgroups smaller than this (day 3 postinfection trials, infected first presentation = 13, uninfected first = 13; day 5 postinfection, infected first = 15, uninfected first = 13; day 7 postinfection, infected first = 15, uninfected first = 12). Beetles were deprived of food for 24h prior to each trial.

**Figure 1:**
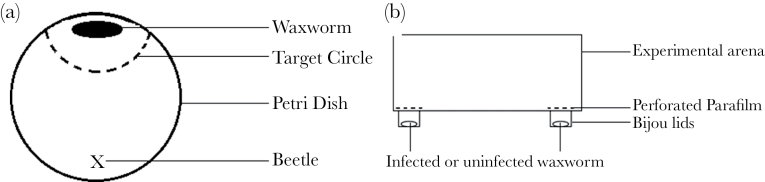
Experimental setup for (a) Experiment 1: petri dish experimental arena with the target area drawn. Infected or uninfected waxworms were placed in the center of the target circle during trials and beetles were moved to position X at the start of each trial and (b) Experiment 2: lateral view of the scent test arena with fresh infected or uninfected waxworms placed in each bijou lid. Opaque Parafilm™ with pierced holes allowed scent to diffuse but no visual signal.

The experimental arena was a petri dish in which a target area was marked with a black marker pen (Figure 1a; a part circle, 3-cm diameter, centered on a position at the edge of the dish). Beetles were given 10min to habituate to the empty dish, then an infected waxworm (day 3, 5, or 7 postinfection, average weight = 0.249g, standard deviation [SD] = 0.016) or an uninfected waxworm larva (average weight = 0.252g, SD = 0.016) was placed in the center of the target area, and an experimental beetle was placed opposite. There was no significant difference in the weight of infected or uninfected waxworms (*W* = 3751.5, degrees of freedom [df] = 79, *P* = 0.2008). Beetles were observed for an hour in a dark room, illuminated by a low-intensity red light to allow observation of the beetles.

We recorded the total duration spent in the target area and time spent feeding (mandibles in contact with the waxworm). To see if chemical repellents affected beetle hygiene behaviors, we also recorded the number of antennal cleans and the total time spent on mandibular cleaning with front legs. For time spent in the target area, timing would not start until the main body of the beetle was within the target; legs only were not counted. After the experiment, the beetles were fed, weighed 1 week later, and then trialed with the reverse condition (those that received uninfected waxworms first, then received infected waxworms and vice versa) at least 1 week after the initial trial.

### Statistical analysis

Data were pooled across the 2 trapping seasons as experiments for different infection stages occurred over both years. Most of the data were left-skewed and conformed reasonably to an exponential distribution and so were analyzed using MCMCglmm in R (Hadfield 2010). Infection status of waxworms was used as a fixed factor, beetle weight and beetle sex as covariates, and order of presentation was included as a random variable, controlling for effects of pseudoreplication. The data for the number of antennal cleans were heavily skewed by zero values for day 7 postinfection data, so a Wilcoxon matched-pairs test (with zero values) was utilized; otherwise, we used an exponential distribution for days 3 and 5. All MCMCglmm analyses were run for 13000 iterations with a thinning interval of 10 iterations. The feeding data for days 3 and 5 postinfection however were not normally distributed and could not be transformed or the appropriate families found in mixed model programs in R. These data were therefore analyzed using a nonparametric Wilcoxon matched-pairs test. The data were further analyzed using a Mann–Whitney test to examine the effect of beetle sex on feeding on uninfected and infected waxworms. The data for the beetles that did not attempt to feed over the 3 infection stages were analyzed with a binomial generalized linear model (glm) using day as a fixed factor. When comparing the infected and uninfected waxworm weights for these trials, the data were not normal and could not be transformed to normal so a Mann–Whitney test was utilized.

There were only 23 cases of mandibular cleaning across all infection stage experiments and so these data were not analyzed.

### Experiment 1: results

**Figure 2:**
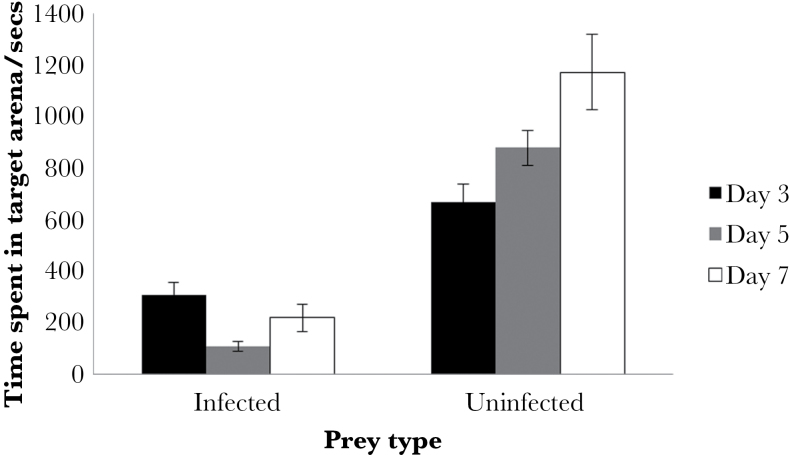
Time spent by *Pterostichus madidus* in a target with either day 3, 5, or 7 *Heterorhabditis bacteriophora*-infected or -uninfected waxworms. Data are shown as means ± standard error.

There was a significant interaction between prey type and beetle sex on the time spent in the presence of infected or uninfected waxworms (Supplementary Figure S3, *P* = 0.004). Female beetles spent less time in the presence of infected and uninfected waxworms than male beetles although the difference was greater when females were presented with uninfected waxworms. Beetles spent more time in the presence of the uninfected than the infected waxworms (Figure 2, MCMCglmm; *P* < 0.001 for all infection stages). Additionally, for beetles receiving infected waxworms 5 days postinfection, there was a prey type × order bias whereby beetles with experience of infected waxworms during their first trial spent more time near uninfected waxworms on their second trial compared with those who had experienced uninfected waxworms on their first trial (MCMCglmm; *P* < 0.001). Comparing time spent near infected waxworms across all 3 infection stages, there was a marginally nonsignificant effect in which beetles spent more time in the target circle with 3-day infected waxworms compared with that spent with days 5 and 7 (MCMCglmm; *P* = 0.062). This indicates that at day 3 of infection the repellent properties of the infected prey may have been less intense than at later stages of infection.

**Figure 3:**
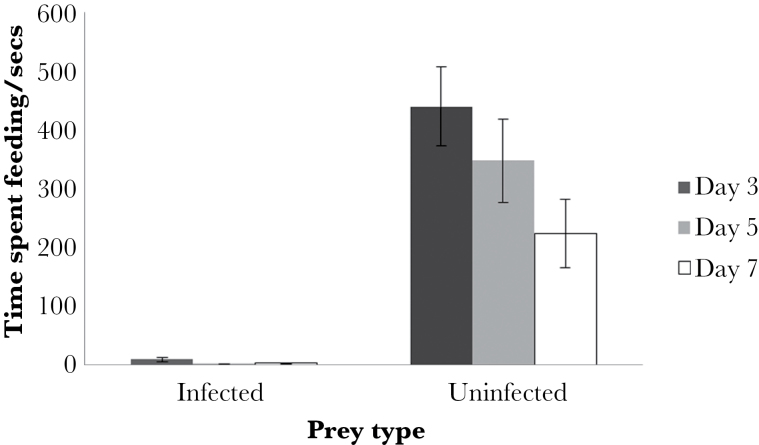
Time spent feeding by *Pterostichus madidus* on either day 3, 5, or 7 *Heterorhabditis bacteriophora*-infected or -uninfected waxworms. Data are shown as means ± standard error.

Beetles spent significantly more time feeding on uninfected waxworms than infected waxworms at each infection stage (Figure 3; MCMCglmm; day 3; *P* < 0.001, day 5; *P* < 0.001, day 7; *P* < 0.001); there was no effect of order of presentation in this test (MCMCglmm; day 3; *P* = 0.644, day 5; *P* = 0.302, day 7; *P* = 0.646) or of sex on day 3 (Mann–Whitney test, uninfected, *P* = 1, infected, *P* = 0.3454) or day 7 (MCMCglmm, *P* = 0.432). However, female beetles spent less time feeding on uninfected waxworms compared with male beetles at day 5 postinfection only (Mann–Whitney test, *P* = 0.01954). However, demonstrating a delay in development of chemical defense, the beetles fed more on day 3 postinfection waxworms than on either day 5 or 7 postinfection waxworms (MCMCglmm, *P* = 0.040). There was no significant difference in time spent feeding on uninfected waxworms across all 3 infection stages (MCMCglmm, *P* = 0.614).

**Figure 4:**
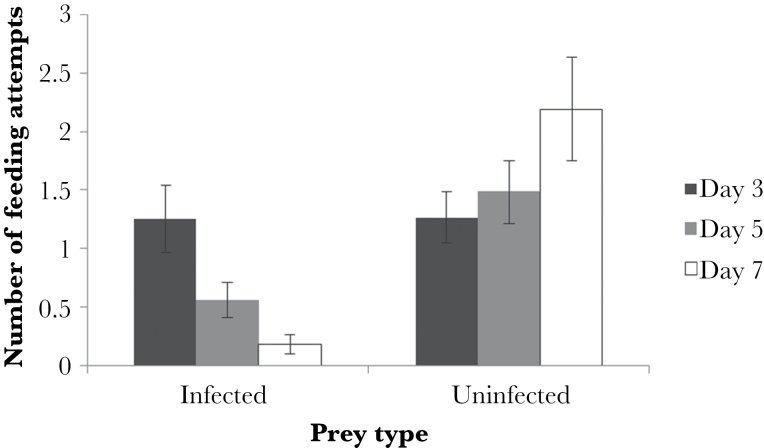
Number of feeding attempts made by *Pterostichus madidus* on either day 3, 5, or 7 postinfection *Heterorhabditis bacteriophora*-infected and -uninfected waxworms. Data are shown as means ± standard error.

Similarly, there was a significant difference in the number of feeding attempts on infected waxworms across the 3 infection stages, with beetles having significantly more feeding attempts on uninfected than infected waxworms at both days 5 and 7 postinfection (Figure 4; MCMCglmm; day 5; *P* = 0.034, day 7; *P* < 0.001). However, and again supporting the view that early infections have little chemical defense, at day 3 postinfection beetles did not have significantly more feeding attempts on uninfected compared with infected waxworms (Figure 4; *P* = 0.258). There was no effect of sex on the number of feeding attempts (MCMCglmm; day 3; *P* = 0.130, day 5; *P* = 0.184, day 7; *P* = 0.424).

Given that vision is not a likely cue for the beetles, there is evidence that odor itself can protect the carcass from attack. Increasing the age of infection significantly increased the proportion of beetles that did not ever feed on the infected host during the trial (3-day postinfection = 38% of beetles; day 5 = 56% of beetles; day 7 = 67% of beetles; binomial GLM: *z* = 2.027, df = 1, *P* = 0.0426). In contrast, only 27.5% of beetles never attacked an uninfected waxworm across all infection stages. However, as the beetles could examine waxworms with their antenna, we could not rule out that some of this avoidance was due to direct chemical assessment, and some due to olfaction. Hence, in the next experiment, we tested the role of olfaction specifically.

Finally, in this experiment, there was no significant difference between the number of antennal cleans performed by *P. madidus* on encountering infected or uninfected waxworms (Supplementary Figure S2; *P* > 0.05 for days 3, 5, and 7 for both the number of antennal cleans [day 3; *P* = 0.750, day 5; *P* = 0.734, day 7; *P* = 0.852] and antennal cleans per se [present or absent] [day 3; *P* = 0.639, day 5; *P* = 0.714, day 7; *P* = 0.208]). Furthermore, there was no effect of beetle sex on the number of antennal cleans performed. However, there was a significant negative effect of beetle weight on the number of antennal cleans performed when beetles were exposed to day 7 postinfection either infected or uninfected waxworms (*F*_1,26_ = 4.609, *P* = 0.041), so that bigger beetles made fewer cleans than smaller beetles.

There were only 6 episodes of mandibular cleaning (beetles utilizing their front tarsi to “wipe” their mandibles) during the day 3 postinfection experiments, 13 during the day 5 postinfection experiments, and 4 during the day 7 postinfection experiments. The time spent mandibular cleaning ranged from 2 to 83s, and the majority of episodes were observed in *P. madidus* that were trialed with infected waxworms.

## EXPERIMENT 2: IS THERE OLFACTORY PROTECTION OF INFECTED WAXWORMS?

This experiment was designed as a 2-choice preference test (Figure 1b). Scent test arenas were created using plastic food containers (Smart Tubz, Tesco, 11cm × 16cm × 4.5cm) with 2 bijou bottle lids (diameter = 15mm, height = 10mm) as scent wells positioned 12cm apart (see Figure 1b). Square pieces of opaque Parafilm™ were then used to cover the scent wells, and 21 holes were pierced with a needle in a grid-like fashion for aeration.

To provide scent cues, 0.3g of macerated fresh infected (either day 3, 5, or 7 postinfection) or fresh uninfected waxworms were measured and put into opposite lids. During an experimental trial, beetles were observed for 1h, and we recorded the time spent in proximity to each scent well. Beetles were tested in 2 trials with the position of the infected waxworm reversed between them (with a minimum of 7 days between first and second trials). Hence, approximately half the beetles (*n* = 10) received scent from uninfected waxworms on the right hand side and the others (*n* = 9) received scent from uninfected waxworms on the left hand side. Arenas were reused between trials but were cleaned with 70% ethanol to prevent beetles leaving olfactory cues to other subjects. Fresh olfactory cues were made on each day of the experiment.

We used the same set of beetles as in Experiment 1, 10 days after the final trial of that experiment; therefore, beetles were experienced predators. As before, beetles were starved for 24h prior to experimentation. Four beetles died after one trial, with exposure to both infected and uninfected scent, and so were removed from the experiment, and 5 beetles died before the experiment started. We again used MCMCglmm (Hadfield 2010) in R, for an exponential distribution. Infection status of waxworms was used as a fixed factor, beetle weight and sex as covariates, and order of presentation was included as a random variable, controlling for effects of pseudoreplication. Data were pooled across both years as olfactory experiments for different infection stages were run across the 2 years.

### Experiment 2: results

**Figure 5:**
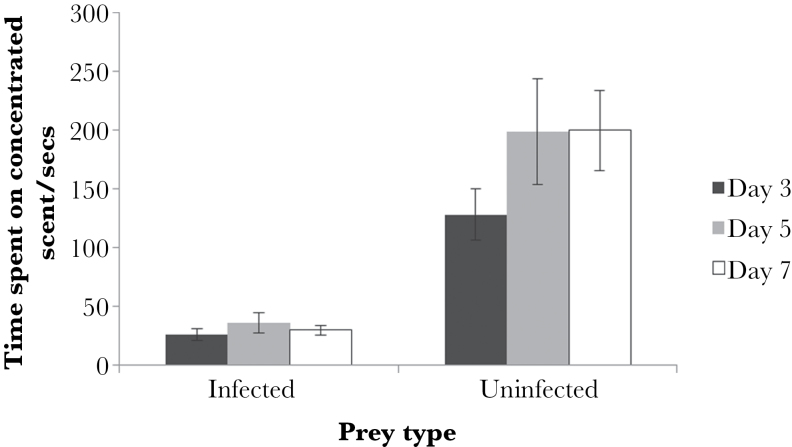
Time spent on either day 3, 5, or 7 *Heterorhabditis bacteriophora*-infected or -uninfected waxworm scent by *Pterostichus madidus*. Data are shown as means ± standard error.

In general, beetles avoided the scent of *H. bacteriophora*-infected waxworms. They spent significantly more time on the uninfected than infected scent across all infection stages (Figure 5; MCMCglmm; day 3; *P* = 0.012, day 7; *P* < 0.001). There was no effect of beetle mass, sex, or order of presentation (i.e., left- or right-side bias) in either the day 3 or 7 test. For the day 5 postinfection scent test, there was a side × prey type bias (MCMCglmm; *P* = 0.034), which showed that beetles spent more time feeding on uninfected waxworms when the infected scent was located on the left hand side of the experimental arena. There was no effect of sex in the day 5 test.

Notably, there was no significant difference in time spent on the infected scent across all 3 infection stages (MCMCglmm; *P* = 0.448). Therefore, beetles showed similar avoidance of the scents of days 3, 5, and 7 postinfection waxworms.

## DISCUSSION

We found that infected carcasses and hence entire colonies of an EPN and its bacterial symbiont are generally protected from attacks by beetles (Experiment 1). Beetles made fewer approaches toward and fewer attacks on waxworms that had been infected with *H. bacteriophora* than those that were uninfected. Furthermore, the carcasses were more vulnerable to attacks by foraging beetles when the infection was young (here 3 days), compared with when it was older (5 or 7 days, Experiment 1). This is consistent with the preferences of foraging birds (robins, Fenton et al. 2011), ants, crickets, and wasps (Gulcu et al. 2012), which also showed less of an aversion to younger infections. Furthermore, females spent less time in the presence of both infected and uninfected waxworms and also less time feeding on uninfected waxworms, which could be due to the trapping schedule with females being less active over summer following egg laying (Matalin 2008).

We demonstrate (for the first time to our knowledge) that olfactory cues from infected carcasses can provide substantial protection, biasing beetles to keep away from hosts infected with an EPN. Hence, we propose that olfaction may work as a first level of defense, an aposematic (warning) signal deterring nocturnal foraging invertebrate predators. A key point here is that the infected carcass does not decay during the infection, rather it is preserved by antimicrobials synthesized by *P. luminescens* (Clarke 2008). Hence, the repellent odor is not that of a decaying corpse, rather it is something conferred by the EPN and/or its symbiont. We suggest that olfaction is a major component of protection of *H. bacteriophora* colonies, and perhaps many other EPNs.

We next considered the results in the context of the functional ecology of the parasite.

### Why “parasite-induced” aposematism?

On the face of it, biosynthesis of a toxin is easy to explain in functional terms. The colony of parasites must survive long enough to reach reproductive capacity where it sends out infectious juveniles. A chemical defense is therefore important to protect the colony by repelling a foraging animal (Baur et al. 1998).

However, we can offer a novel perspective on this question. Shortly after infection by *H. bacteriophora*, the symbiotic bacterium (*P. luminescens*) kills the host (Clarke 2008) in order to protect itself from the host’s highly effective immune defenses, to which these parasites are known to be susceptible (Goodrich-Blair and Clarke 2007). On the one hand then, killing the host removes the threat from immune defenses, but on the other hand, a dead host cannot defend its parasites against predators by its normal behaviors of escape, retaliation, and secretion of noxious substances (Eisner et al. 2005). We propose that bacterial toxin synthesis is essential to EPN infections as a replacement to the host’s original antipredator defenses.

In effect then, the parasites kill the host in order to shut down its immune defenses, but then have to replace its now defunct antipredator defenses with a suite of its own protections, including toxicity and in all likelihood aposematic signaling. If this interpretation is correct, then we can predict that nematode infections that do not kill their hosts would then be less likely to synthesize toxins de novo.

### Protection of early infections

Our results show that new infections (here at least 0–3 days) are vulnerable to beetle foragers (no difference in feeding attempts between uninfected and infected waxworms at day 3), which ties in with other results showing vulnerability of early infections to birds Fenton et al. (2011) and other arthropods Gulcu et al. (2012). We found no material increase in protection between days 5 and 7, however, suggesting that antiforager defense levels reach some plateau around day 5. We can now ask a number of questions about the vulnerability of early stage infections.

First, are there any compensating defenses that can act earlier than toxicity to protect the new, vulnerable colony? In our olfaction experiment (Experiment 2), the beetles spent a similarly small amount of time in proximity to the infected host cue regardless of the stage of infection. This suggests that in the right conditions, specifically where the forager has a choice to orient to an alternative “good” food source, olfactory cues can be effective for young infections. Hence, olfactory protection may build up quickly and may be one way that young colonies can limit their exposure to risk. If olfaction provides protection even without a chemical defense, this suggests that the olfactory component may be aversive in itself to foraging arthropods, as well as functioning as an aposematic cue, later warning predators of toxicity (see discussion in Rowe and Halpin 2013).

A second question is why, even 3 days after infection the protection of the host carcass is relatively poor. Answering this question requires knowledge of the risk to the host per unit time as well as the constraints on bacterial toxin synthesis. Perhaps the simplest explanation is then that the bacterial population takes several days to reach the size necessary to produce sufficient toxin to protect the prey. Perhaps also the threat per day to a colony within a host is small, so poor protection for 3 days poses only a small threat provided the colony is then well protected for the next 17 days or so. An interesting question then is what are the trade-offs within the bacterium *P. luminescens*, which determine relative investment in defensive toxins, olfactants, and pigmentations? This is currently unknown, but key to understanding the development of defense in this (and other EPN) species. We note that the functional genetics of *P. luminescens* defenses are relatively well understood (Clarke 2008; Waterfield et al. 2009); hence, this is an exciting opportunity for further work.

A third question is whether there are any other lines of protection of early infection that we have not considered. We note also, but have not been able to test the possibility that the short period of bioluminescence that is observable in *H. bacteriophora* infections could operate as an aposematic cue protecting new infections (Waterfield et al. 2009). Wild toads (*Bufo bufo*) have been shown to lower attack rates and increase latencies toward bioluminescent glow-worm larva (Coleoptera: Lampyridae) in their native range (De Cock and Matthysen 1999, 2003). This bioluminescence is generated by the bacterial symbiont *P. luminescens* shortly after host death, lasting between 24- and 36-h postinfection. It is therefore focused into the period in which, from our data, chemical defense is lacking. The evolutionary reason for its persistence is obscure. On the one hand, it could be a nonfunctional by-product of bacterial metabolism (Waterfield et al. 2009), but on the other, it could function to protect young infections from attack. Beetles and other invertebrates are possible targets of such putative bioluminescent aposematic signaling. However, their limited vision may inhibit the value of this trait as a defense. If bioluminescence in *H. bacteriophora* infections is effective in protecting colonies of EPNs, we suggest it is by deterring small nocturnal mammals from attack; this possibility remains to be tested.

### Evolution of infectious aposematism

In our view, there is good and growing evidence that EPNs may protect themselves by what we term “parasite-induced aposematism.” We have shown that chemical defense develops over the first few days of infection and appears to reach a stable level by 5-day postinfection. Furthermore, olfactory cue(s) associated with infection repel foragers and protect colonies from a nocturnally active invertebrate.

There may then be 3 forms of aposematic signal in this system: bioluminescence, olfaction, and red pigmentation. Each putative signal may apply to different kinds of enemy: bioluminescence to nocturnal, visually capable mammals (De Cock and Matthysen 2003); olfaction to nocturnal (and perhaps diurnal) foragers, including numerous visually limited invertebrates; epidermal color change to diurnally foraging animals such as birds. Hence, the “multimodal” nature of the defense in EPNs may be attributable to the wide range of enemies encountered (Rowe and Halpin 2013).

Given the wide range of enemies, we can ask—would it not be better for the colony to allocate all of its defensive resources to one generally acting defense of heightened intensity? For example, why does the colony within the host not just invest its defensive resources only in toxicity, to make the carcass even more toxic and repellent? One reason is that toxicity is the last line of defense, only operating when the forager has made contact with the host carcass, and is in a position to damage it and the parasites inside. Some foragers may do substantial damage before coming into contact with and being repelled by the defensive toxins. Hence, it is especially important for the parasite that it develops and deploys cues, which act at a distance, and given the different sensory specializations of the potential enemies, we can expect a multimodal set of cues.

## CONCLUSIONS

Our work adds further substance to the hypothesis of Fenton et al. (2011) that EPNs cause “parasite-induced aposematism” in their host carcasses. This provides the dual protection from a potent chemical defense and from advertisement of that defense by some kind of warning display. We propose here that olfactory cues may be an important component of that warning display, perhaps the only one available to some classes of predator, such as nocturnally hunting arthropods. Olfaction may also be quick to develop, and provide a repellent defense in itself while repellent toxins build up inside the dead host. Though recognized for some years (Baur et al. 1998), we contend that the defensive adaptations of EPNs are a fascinating and understudied component of their life history patterns.

## SUPPLEMENTARY MATERIAL

Supplementary material can be found at http://www.beheco.oxfordjournals.org/

## FUNDING

We thank Natural Environment Research Council (NE/K500975/1) for funding for this project.

## Supplementary Material

Supplementary Data
