# Supplementary material for: “Parasite-induced aposematism” protects entomopathogenic nematode parasites against invertebrate enemies
Source: Behav Ecol. 2015 Nov 27;27(2):645–51. doi: 10.1093/beheco/arv202 (PMC4797382; doi:10.1093/beheco/arv202)
Supplement: Supplementary Data [file supp_27_2_645__index.html]

“Parasite-induced aposematism” protects entomopathogenic nematode parasites against invertebrate enemies — “Parasite-induced aposematism” protects entomopathogenic nematode parasites against invertebrate enemies — Supplementary Data 

# “Parasite-induced aposematism” protects entomopathogenic nematode parasites against invertebrate enemies

## Supplementary Data

Data files

- Supplementary Data - Supplementary Data
- Supplementary Data - Supplementary Data
- Supplementary Data - Supplementary Data
