# Supplementary figures and images for: “Parasite-induced aposematism” protects entomopathogenic nematode parasites against invertebrate enemies
Source: Behav Ecol. 2015 Nov 27;27(2):645–51. doi: 10.1093/beheco/arv202 (PMC4797382; doi:10.1093/beheco/arv202)

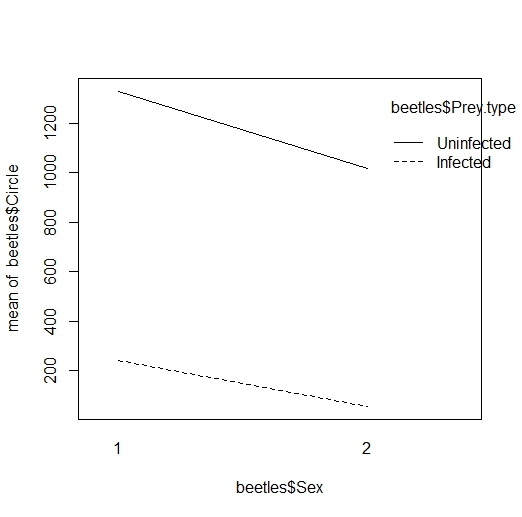

Supplement: Supplementary Data [file supp_arv202_Supplementary_20S3.tif]

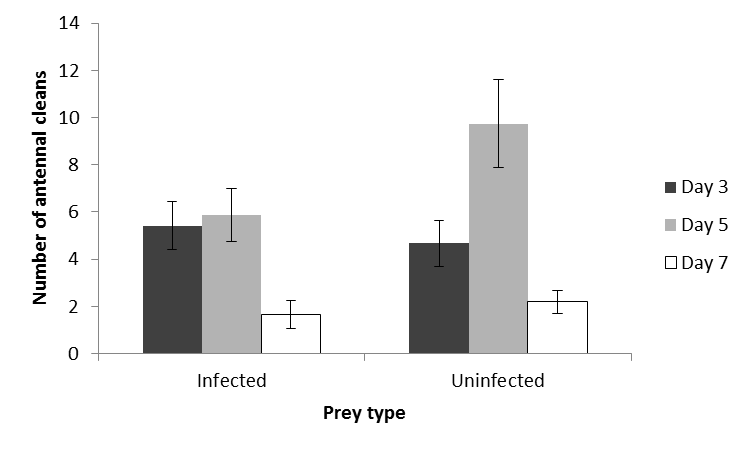

Supplement: Supplementary Data [file supp_arv202_Supplementary_20S2.tif]
