# Supplementary material for: “Parasite-induced aposematism” protects entomopathogenic nematode parasites against invertebrate enemies
Source: Behav Ecol. 2015 Nov 27;27(2):645–51. doi: 10.1093/beheco/arv202 (PMC4797382; doi:10.1093/beheco/arv202)
Supplement: Supplementary Data [file supp_arv202_Supplementary_material.docx]

**Supplementary material:**

Table S1. Number of male and female beetles utilised during experiment 1. Beetles were sexed via dissection following the trials.

| Day of Experiment | Number of Males | Number of Females |
| --- | --- | --- |
| 3 | 17 | 9 |
| 5 | 14 | 13 |
| 7 | 22 | 5 |

Figure S2. Number of antennal cleans performed by *P. madidus* when encountering either day 3, 5 or 7 *H. bacteriophora-*infected or -uninfected waxworms. Data are shown as means ± SE.

Figure S3. Interaction plot between beetle sex, prey type and time spent in the circle.
